# The diagnostic accuracy of serum microRNAs in detection of cervical cancer: a systematic review protocol

**DOI:** 10.1186/s41512-023-00142-4

**Published:** 2023-01-31

**Authors:** Frank Ssedyabane, Nixon Niyonzima, Joseph Ngonzi, Deusdedit Tusubira, Moses Ocan, Dickens Akena, Eve Namisango, Robert Apunyo, Alison Annet Kinengyere, Ekwaro A. Obuku

**Affiliations:** 1 Department of Medical Laboratory Science, Mbarara University of Science of Science and Technology, P.O. Box 1410, Mbarara, Uganda; 2 The Uganda Cancer Institute, P. O. Box 3935, Kampala, Uganda; 3 Department of Obstetrics and Gynaecology, Faculty of Medicine, Mbarara University of Science of Science and Technology, P.O. Box 1410, Mbarara, Uganda; 4 Department of Biochemistry, Mbarara University of Science of Science and Technology, P.O. Box 1410, Mbarara, Uganda; 5 Africa Centre for Knowledge Translation and Systematic Reviews, College of Health Sciences, Makerere University, P.O. Box 7072, Kampala, Uganda; 6 Department of Pharmacology & Therapeutics, College of Health Sciences, Makerere University, P.O. Box 7072, Kampala, Uganda; 7 Department of Psychiatry, College of Health Sciences, Makerere University, P.O. Box 7072, Kampala, Uganda; 8 Sir Albert Cook Medical Library, Makerere University, College of Health Sciences, P.O Box 7072, Upper Mulago Hill Road, Kampala, Uganda; 9 Clinical Epidemiology Unit, Department of Medicine, School of Medicine, College of Health Sciences, Makerere University, P.O. Box 7072, Kampala, Uganda

**Keywords:** Cervical cancer, Serum microRNAs, Diagnostic utility, Cervical intraepithelial neoplasia, Systematic review

## Abstract

**Background:**

Cervical cancer remains a public health problem worldwide, especially in sub-Saharan Africa. There are challenges in timely screening and diagnosis for early detection and intervention. Therefore, studies on cervical cancer and cervical intraepithelial neoplasia suggest the need for new diagnostic approaches including microRNA technology. Plasma/serum levels of microRNAs are elevated or reduced compared to the normal state and their diagnostic accuracy for detection of cervical neoplasms has not been rigorously assessed more so in low-resource settings such as Uganda. The aim of this systematic review was therefore to assess the diagnostic accuracy of serum microRNAs in detecting cervical cancer.

**Methods:**

We will perform a systematic review following the Preferred Reporting Items for Systematic Review and Meta-Analysis Protocols (PRISMA-P) statement. We will search for all articles in MEDLINE/PubMed, Web of Science, Embase, and CINAHL, as well as grey literature from 2012 to 2022. Our outcomes will be sensitivity, specificity, negative predictive values, positive predictive values or area under the curve (Nagamitsu et al, Mol Clin Oncol 5:189-94, 2016) for each microRNA or microRNA panel. We will use the quality assessment of diagnostic accuracy studies (Whiting et al, Ann Intern Med 155:529-36, 2011) tool to assess the risk of bias of included studies. Our results will be reported according to the Preferred Reporting Items for Systematic Reviews and Meta-Analysis for Diagnostic Test Accuracy studies (PRISMA-DTA). We will summarise studies in a flow chart and then describe them using a structured narrative synthesis. If possible, we shall use the Lehmann model bivariate approach for the meta analysis

**Use of the review results:**

This systematic review will provide information on the relevance of microRNAs in cervical cancer. This information will help policy makers, planners and researchers in determining which particular microRNAs could be employed to screen or diagnose cancer of the cervix.

**Systematic review registration:**

This protocol has been registered in PROSPERO under registration number CRD42022313275.

## Background

Worldwide, there were an estimated 770,828 incident cervical cancer cases in 2020 [1]. Cervical cancer is the second most common cancer among women aged 30 to 45 years of age worldwide [2]. It has been reported that cervical cancer accounts for more than 270 000 deaths annually, 85% of which occur in developing countries [3] especially in sub-Saharan Africa [4, 5]. Cervical Cancer stands at 43/100,000 cancer cases in East Africa [6], and in Uganda over 4,000 new cases are recorded annually and 58% of these result into death [7, 8]. A well-proven way to prevent cervical cancer is to screen and detect pre-cancers before they can turn into invasive cancer [3]. Cervical cancer is a very treatable disease when diagnosed early before advancement [6]. However, only 5% of women in low and middle-income countries undergo cervical cancer screening [8]. The current approaches to cervical cancer screening and diagnosis include visual inspection with acetic acid, Pap smear cytology, colposcopy, and histology. However, Pap smear, the most widely used screening method, is limited by its low accuracy (sensitivity and specificity), compared with newer DNA-based methods, especially in identifying cancer in dysplastic squamous and glandular cells of the cervix. New methods of cervical cancer screening that are less costly and user-friendly suit for a developing country’s contexts [8].

### The research gap

There are a number of biomarkers that have been studied, many of which are associated with cervical cancer or cervical intraepithelial neoplasia, but yet to be evaluated as potential early indicators for cancer or cervical intraepithelial neoplasia. MicroRNAs are easily quantified in blood and standardised laboratory methods can easily be developed for their quantification. MicroRNAs belong to a novel category of small non-coding RNA molecules that interconnect to target mRNA to either degrade or modify it [9]. They catalyse mRNA cleavage by inhibiting its translation processes [10]. They have 22 to 24 nucleotides [11, 12]. MicroRNAs were previously thought to originate from tumour cells, but currently, it is known that they can exist in body fluids, especially blood [13, 14]. Recent evidence suggests that exosomal microRNAs in liquid biopsies like blood have the potential to improve prognostic and diagnostic workup in cancer [15].

Specifically for cervical cancer Allegra et al. [16] and Anindo and Yaqinuddin [17] revealed that microRNAs are expressed both in cancerous tissues [18] and in serum [19]. As a result, serum concentrations of microRNAs have been proposed as diagnostic and prognostic monitoring tools for cancer [20]. Evidence strongly points at microRNAs being prognostic of cervical cancer [9, 19, 21–28]. Clearly, microRNAs are the future of cervical cancer screening and diagnosis. Several studies on premalignant lesions indicate that microRNAs are involved at every stage during the development of invasive cervical cancer [29–33]. Multiple studies have shown that a number of microRNAs are upregulated during the progression to cervical cancer [34]. For instance, miR10a has been shown to have an increased expression during the development of cervical cancer [35–37] as well as miR20b [35, 37, 38], miR9 [35, 37, 38], miR16 [35, 36, 38] and miR106a [35, 36, 38]. From a systematic review by Gao et al [34], miR16, miR106a and miR21 are equally upregulated and are associated with progression from intermediate stages to cervical cancer. MiR21 has specifically been implicated by a number of studies to be upregulated during cervical carcinogenesis [35, 39–42].

Different authors however report different sensitivity and specificity values for different microRNAs in respect to cervical cancer detection. In a view of having new non-invasive, user-friendly, accurate, and standardisable tests, it is necessary to conduct a systematic review, to assess the diagnostic accuracy of different serum microRNAs in the detection of cervical neoplasms. This systematic review will therefore determine the diagnostic accuracy of individual serum or plasma microRNAs or microRNA panels in detecting cervical intraepithelial neoplasia or cervical cancer in women of reproductive age globally.

## Methods

We developed a protocol a priori following the Preferred Reporting Items for Systematic Review and Meta-Analysis Protocols (PRISMA-P) recommendations [43] and registered it in the PROSPERO database, number CRD42022313275. In drafting the final report, we will follow the Preferred Reporting Items for Systematic Reviews and Meta-analysis for Diagnostic Test Accuracy (PRISMADTA) [44, 45].

### Review question

What is the diagnostic accuracy of individual serum/plasma microRNA or microRNA panels in detecting cervical cancer compared to histology (gold standard) among women of reproductive age worldwide?

### Eligibility criteria

#### Study design eligibility criteria

We shall include all prospective and retrospective cohorts, cross-sectional, and case-control that report diagnostic accuracy of serum microRNAs or panels of microRNAs in the detection of cervical intraepithelial lesions or cervical cancer. Also, in the meta-analysis, we shall include those diagnostic test accuracy studies that adhered to the STARD (Standards of Reporting Diagnostic Accuracy Studies) [46]. We will exclude all studies done on nonhuman participants or those that did not report measures of diagnostic accuracy as required by STARD.

#### Population and condition under study

We will include all those studies that quantified serum microRNAs in symptomatic or asymptomatic women aged 18 years and above globally, as a diagnostic or screening test for cervical intraepithelial neoplasia or cervical cancer (Table 1).

**Table 1:** Inclusion criteria

Study characteristics	Inclusion criteria
Population	Women aged 18 years and above that are being investigated for cervical cancer or cervical intraepithelial lesions.
Index tests/exposure	Serum microRNA concentrations
Comparison	Histological grades of cervical cancer or cervical intraepithelial lesions.
Outcomes	Sensitivity, specificity, negative predictive values or positive predictive values
Study designs	All prospective and retrospective cohorts cross-sectional and case-control studies that reported measures of diagnostic accuracy.
Time duration	2012–2022

#### Exposure/index tests

We will include those studies that quantified blood-based microRNAs from women receiving cervical cancer screening/diagnostic services (Table 1).

#### Comparison

The comparison gold standard or reference test will be histological results (grades) for cervical intraepithelial neoplasia or cervical cancer. No any other tests like clinical assessment will be considered (Table 1).

#### Outcomes

Our outcome will be measures of diagnostic accuracy (sensitivity, specificity, negative predictive values, or positive predictive values) for each reported microRNA (Table 1).

#### Study design

We shall include observational studies amenable to diagnostic accuracy studies, mainly cross-sectional, case-control as well as experimental designs like randomised trials, cluster randomised trials and quasi-experimental designs (Table 1).

#### Timeframe

We shall include primary studies carried out within a 10-year period, from 2012 to 2022 (Table 1).

### Search strategy

#### Data sources

The data sources will include databases, institutional websites, grey literature and contacting authors. To identify all the studies, we will search MEDLINE through the PubMed platform, Web of Science, Embase through the Ovid platform, CINAHL, and Scopus. We will also search for grey literature such as conference papers, technical reports, theses, and dissertations in Google Scholar, Google, OpenGrey, ProQuest Dissertations & Theses, and British Library EThos. The authors will search each database from 2012 to 2022.

We will also screen through reference lists of included studies for additional eligible studies that may not be identified by the search. Systematic reviews will also be used to identify additional primary studies.

#### Electronic search

The electronic search will explore the combinations of the keywords covering the PICOS elements. The population component will include the words “Uterine cervical neoplasms*” [Mesh] OR “Cervical cancer*” [tw] OR “Human uterine cancer*” [tw] OR “SCC” [tw] OR “Cancer of the cervix*” [tw] OR “Cervical intraepithelial neoplasia*” [tw] OR “CIN” [tw].

The intervention component will include: “Circulating MicroRNA” [Mesh] OR “Circulating microRNAs*”[tw] OR “Circulating miRNAs” [tw] OR “Circulating serum microRNAs*”[tw] OR “Circulating serum miRNAs*”[tw] OR “Serum microRNAs*”[tw] OR “Serum miRNAs*”[tw] OR “Biomarkers*”[tw] OR “Blood*”[tw] OR “microRNAs*”[tw].

For the comparator, there will be no specific terms since they are already considered in the description of the population.

The outcome component will include the words “Early Detection of Cancer*” [Mesh] OR Diagnosis*[tw] OR “Diagnostic value*” [tw] OR “Diagnostic utility*” [tw] OR Sensitivity* [tw] OR Specificity*[tw] OR Specific*[tw] OR Sensitive*[tw] OR “up regulated*” [tw] OR “Down regulated*” [tw] OR “increased*” [tw] OR “Decreased*” [tw] OR “positive predictive value*” [tw]. OR “Negative predictive value*” [tw].

We will not include specific study designs in the search. We shall instead apply this in the eligibility criteria. The full search string is available in Table 2.

**Table 2:** Feasibility of yield of literature of pilot electronic search strategy for diagnostic utility of Serum microRNAs in detection of cervical intraepithelial neoplasia and cervical cancer

Search number (Data base)	Search terms (and date)	Number of hits (relevant)
#1(PubMed)	Search: ((“Uterine cervical neoplasms*” [Mesh] OR “Cervical cancer*” [tw] OR “Human uterine cancer*” [tw] OR “SCC” [tw] OR “Cancer of the cervix*” [tw] OR “Cervical intraepithelial neoplasia*” [tw] OR “CIN” [tw].) AND (“Early Detection of Cancer*”[Mesh] OR Diagnosis*[tw] OR “Diagnostic value*” [tw] OR “Diagnostic utility*” [tw] OR Sensitivity* [tw] OR Specificity*[tw] OR “up regulated*” [tw] OR “Down regulated*” [tw] OR “increased*” [tw] OR “Decreased*” [tw].)) AND (“Circulating MicroRNA” [Mesh] OR “Circulating microRNAs*”[tw] OR “Circulating miRNAs” [tw] OR “Circulating serum microRNAs*”[tw] OR “Circulating serum miRNAs*”[tw] OR “Serum microRNAs*”[tw] OR “Serum miRNAs*”[tw] OR “Biomarkers*”[tw] OR “Blood*”[tw] OR “microRNAs*”[tw])	^a^5333 (26, 0.26%)^b^

^a^Number of article titles and abstracts as of 25 April 2022

^b^Sorted by relevance and initial screening of titles and abstracts

We will combine keywords, MESH terms and their synonyms, and these will be divided into three components. All the search components will be combined with the Boolean operators “AND” while the keywords within each component will be combined with “OR.” There will be no language restrictions for this review. We will run the searches just before the final analyses to retrieve the most recent studies eligible for inclusion.

To assess the feasibility of this review, we piloted this search in PubMed and it yielded 5,333 titles and abstracts. We sorted these by relevance and screened the first 500 for which we considered 26 (1%) as potentially eligible for data extraction (Table 2).

#### Selection of studies

Two reviewers (FS and RA) will perform duplicate and independent data extraction. Screening will be a two-step process with initial title/abstract screening followed by retrieval of full-texts and their screening. In any case, a discrepancy will be solved by either a third reviewer (EAO) or by consensus. We will provide a list of excluded full-text articles with reasons for exclusion as an appendix of the final report.

#### Data items, extraction, and management

We will develop a data extraction form, and this will be piloted initially to achieve a good level of agreement between the data extractors. Two reviewers (OM and AD) will independently extract data from all eligible articles. The following data will be extracted:

- *Study characteristics*: author, year of publication, country, study design, sample size, clinical/study setting, number of dropouts with reason, and funding source.
- *Population characteristics*: inclusion/exclusion criteria and patient demographics such as age as well as comorbidities.
- *Laboratory testing*: index testing method, type of sample (e.g. whole blood, serum or plasma), and units of measurement. Type of microRNA(s) studied
- *Gold standard*: Histological confirmation or rule out of cancer.
- *Outcomes*: sensitivity, upregulated, downregulated, increased, decreased, specificity, the predictive value of positivity and negativity.

We will also extract and report the 2×2 tables (describing true and false positives and negatives) from each included study.

#### Risk of bias and methodological quality assessment

Two researchers will independently assess for the risk of bias using the quality assessment of diagnostic accuracy studies [47]. This widely recognised tool evaluates the risk of bias of diagnostic test accuracy research across four domains including patient selection, index test, reference standard and flow and timing. Application of this tool involves summarising the review question, tailoring the tool to the review, generating review-specific guidelines, constructing a flow diagram for each primary study and finally assessing the risk of bias and other concerns regarding applicability.

#### Minimising bias in selection and extraction of data from included studies

A second reviewer (AAK) will validate the electronic search by performing a second and independent search in PubMed using the same search strategy. The second reviewer will also screen all articles that will be excluded by the first pair of reviewers. We will resolve any disagreements among reviewers during screening, selection, abstraction, and risk of bias assessment through consensus or third-party reviewer (EAO) where the need arises.

#### Minimising publication bias

By including both published and unpublished data from multiple sources, we shall be able to minimise publication bias. However, we shall investigate for publication bias by visually inspecting a funnel plot, but only when there are more than 10 studies. We shall not search trial registries for unpublished diagnostic accuracy studies.

#### Statistical analyses and evidence synthesis

An overview of the available studies will be summarised in the flow chart and tabulated. We will describe data from eligible studies in a structured narrative synthesis. It is in this narrative synthesis that we will summarise the article author, year of publication, setting, study designs, sample size and population, type of laboratory index and reference tests, and diagnostic test accuracy outcomes.

Otherwise, we will use the Lehmann model bivariate approach for the meta-analysis. This will only be conducted if a given microRNA type was reported by more than two studies. We will derive summary receiver operating characteristic curves (SROC) for those specific microRNAs using the diagnostic odds ratio as the main outcome measure. We will also derive pooled sensitivity, specificity as well as areas under the curve (AUCs) for those specific microRNAs.

#### Heterogeneity assessment

We will inspect forest plots to initially assess heterogeneity and then check for individual study results in the ROC space. We expect potential sources of heterogeneity to include the year of publication, the country where the study was carried out, intervention types, and outcome measures. Heterogeneity will be statistically quantified using the *I*^2^ statistic and tested for significance using Cochran’s Q.

#### Handling of missing data

For variables that are needed but found either missing or not reported, we plan to label them as not reported, “NR”. Thereafter, we shall seek clarification from the authors on a case-by-case basis. We do not intend to apply any secondary analyses on such missing data.

#### Reporting the review findings

We will report the findings of this review in line with the PRISMA statement. The first table will summarise the author, publication year, study designs, participants, microRNA test details, sample types (serum or plasma) and the gold standard (reference test) used. These will be histological grades of cervical cancer or cervical intraepithelial lesions. The second table will capture the observed outcomes for each microRNA. These will include sensitivity, specificity, negative predictive value, positive predictive value, increased/upregulated, decreased/downregulated or unaffected.

#### Confidence in cumulative evidence

The review authors shall employ the Grading of Recommendations, Assessment, Development and Evaluation (GRADE) approach to rate the certainty of evidence in primary studies. This will be done by considering the risk of bias and performing heterogeneity tests. Then we shall rate evidence as high, moderate low or very low.

#### Knowledge translation

To begin with, we will publish a scientific paper in an open access peer-reviewed and indexed journal relevant to the field of cervical cancer diagnostics. Also, the authors will attend and present results from this review at both local and international scientific conferences. We will engage relevant stakeholders including decision makers, patient groups, oncologists, researchers and PhD students who are currently studying microRNAs in cervical cancer, to deliberate on the policy options.

#### Preliminary findings

We have tabulated five example studies that potentially meet our eligibility criteria. All were identified from our PubMed search (Table 3). These studies were published between 2014 and 2021, all conducted in China. All these studies were case-control designs and addressed the diagnostic accuracy of serum microRNAs in cervical cancer or cervical intraepithelial neoplasia.

**Table 3:** Preliminary results of eligible studies

Author	Year	Country	Design	microRNA studied	AUC	Sensitivity %	Specificity%
Ge et al.	2019	China	Case-control	MicroRNA1290	0.796	90.3	60.2
Qianqian et al	2016	China	Case-control	hsa-mir-92a	0.83	69.6	80.4
Hu et al.	2017	China	Case-control	MicroRNA145	0.848	81.7	63.3
Zenta et al.	2021	China	Case-control	MicroRNA100	0.879	91.2	80.4
Yumei et al.	2021	China	Case-control	MicroRNA18a	0.856	95.2	75.7

## Discussion

Cervical cancer is a big health challenge all over the world, but much of it is felt in low and middle-income countries. The good news is that it can be dealt with once diagnosed at an early stage, i.e. when still at the stage of cervical intraepithelial neoplasia. This calls for accurate and user friendly tests that are easily taken up by both the patients and the health care providers. MicroRNAs are the best candidates to save the world from such a dilemma. What we do not know, however, is the diagnostic accuracy of these microRNA and which exact ones can be used by health care providers.

By answering these questions, we will have a better understanding of where these microRNAs could fit in the current screening/diagnostic strategies for cervical cancer. In particular, these microRNAs could be used as stand-alone screening tests or in conjunction with others, in a new algorithm, or together with the existing ones. This information will be of value to policy makers, planners and researchers in determining which ones and how these microRNAs could be employed to screen for cancer of the cervix.

## Data Availability

All the data generated from this review will be available from the corresponding author upon meaningful request.

## Abbreviations

CIN: Cervical intraepithelial neoplasia
microRNA: Micro ribonucleic acid
Pap: Papanicolaou test
PCR: Polymerase chain eeaction
VIA: Visual inspection with acetic acid
WHO: World Health Organization
